# Synergistic Effects of Using Trace NbB_2_ and Ti Additions to Refine the Microstructure and Enhance the Mechanical Properties of PDC Al-Si Alloy

**DOI:** 10.3390/ma19112227

**Published:** 2026-05-25

**Authors:** Wenxue Fan, Zhuo Zhang, Zengshuo Zhang

**Affiliations:** 1 School of Mechanical and Automotive Engineering, Ningbo Institute of Technology, Ningbo 315336, China; 2 Ningbo Research Institute of Dalian University of Technology, Ningbo 315016, China; zhangz_nbi@dlut.edu.cn; 3 School of Materials Science and Engineering, Dalian University of Technology, Dalian 116024, China; zzs88@mail.dlut.edu.cn

**Keywords:** die-cast Al alloy, grain refiner, microstructure evolution, mechanical properties

## Abstract

Grain refinement is a crucial technological strategy for achieving microstructural homogenization and enhancing the mechanical performance of aluminum alloys. This study examines the synergistic effects of trace additions of NbB_2_ and Ti on microstructural refinement and the enhancement of mechanical properties in high-pressure die-cast (HPDC) Al-Si series alloys. Through systematic investigations utilizing scanning electron microscopy (SEM), electron backscatter diffraction (EBSD), and transmission electron microscopy (TEM) analyses, the mechanisms by which trace additions of NbB_2_ and Ti contribute to the synergistic refinement and strengthening of mechanical properties in HPDC Al-Si alloys are elucidated. The incorporation of NbB_2_ effectively refines both the externally solidified crystals (ESCs) and secondary α-Al_II_ grains. The combined addition of NbB_2_ and Ti further amplifies this effect, resulting in optimal refinement outcomes in the Al-Si series alloy, characterized by an ESC grain size of 19.1 μm and an area fraction of 27.5%, as well as an α-Al_II_ grain size of 6 μm. TEM observations reveal the formation of a Ti-rich transition layer between NbB_2_ and Al with the synergistic addition of Ti and NbB_2_, which fills interface nucleation vacancies and enhances refinement performance.

## 1. Introduction

In recent years, a global consensus on the importance of energy conservation and environmental protection has catalyzed the increased utilization of lightweight materials within the automotive sector. To facilitate weight reduction, there is a growing trend towards the manufacturing of automotive components using lightweight materials, accompanied by a reduction in the wall thicknesses of structural parts [1]. High-pressure die casting (HPDC) presents significant advantages in the production of thin-walled components, ensuring high production efficiency. This process entails the rapid injection of molten metal into a metal mold cavity, achieving a cooling rate of approximately 500 K/s to promote rapid solidification [2]. However, the rapid injection process is intrinsically linked to issues of gas entrapment. Trapped gases expand at elevated temperatures, resulting in the formation of bubbles that complicate the heat treatment of many castings [3]. As a result, untreated aluminum alloys have been the subject of extensive research and application as HPDC thin-walled components.

The Al-Si series alloys are particularly prevalent in automotive, aerospace, and electronic applications due to their high production efficiency, good dimensional accuracy, and excellent formability [4,5]. The die-casting process, characterized by high-speed filling and rapid solidification, often results in complex microstructures that can lead to defects such as segregation, porosity, and cold shuts, thereby significantly constraining the enhancement of mechanical properties [6,7]. The microstructure of die-cast alloys, particularly the externally solidified crystals (ESCs) and secondary aluminum grain structures, plays a crucial role in determining their mechanical properties and has thus become a central focus of research in casting metallurgy in recent years [8,9].

The ESC structure pertains to the partially or fully solidified formations that occur prior to the alloy’s entry into the mold cavity for final solidification. These structures are typically observed in the inner gating region, thick-walled sections, and hot spots. During the die-casting process, these structures cannot be remelted or adequately nucleated, often resulting in coarse grains, non-uniform composition distribution, and pronounced inheritance characteristics, which adversely affect the uniformity and performance stability of the final casting [10,11]. Coarse equiaxed solidification structures (ESCs) can diminish the grain boundary strengthening effect and undermine the fine-grain strengthening mechanism. The pronounced structural disparities between these ESCs and the surrounding normally solidified structures may result in stress concentration, micro-crack propagation, and interphase debonding [3].

Consequently, grain refinement in die-cast alloys is of paramount importance. Numerous effective grain refiners have been investigated for refining die-cast aluminum alloys. For example, titanium (Ti), strontium (Sr), and rare earth elements (RE) have been shown to refine aluminum alloys through the growth restriction effect [12,13,14]. Additionally, the incorporation of grain refiners containing TiB_2_ particles, Al_4_C_3_ particles, or other intermetallic compounds has demonstrated a significant refining effect [15,16]. The practice of adding grain refiners with effective nucleating particles to regulate the nucleation process has become prevalent in the casting industry. The Al-Ti-B series grain refiners, which contain both Al_3_Ti and TiB_2_ particles, are recognized as the most widely used grain refiners for aluminum alloys [17,18]. The excessive presence of silicon (Si) in Al-Si series alloys, particularly when the Si content exceeds 5 wt.%, adversely impacts the grain refinement efficacy of Al-Ti-B grain refiners, a phenomenon commonly termed the “poisoning” effect. The efficacy of Al-Ti-B refiners is reduced due to the formation of Ti-Si intermetallic compounds (e.g., TiSi_2_) on the grain refiner particles, which inhibits heterogeneous nucleation of α-Al [19].

In response to this detrimental “Si poisoning” phenomenon, considerable research efforts have been directed towards developing alternative grain refiners. Among these innovations is the Al-Nb-B grain refiner, which has demonstrated significant grain refinement capabilities in Al-Si-based alloys [20,21,22,23]. More recently, Xu et al. [24] reported that (Nb, Ti)B_2_ compounds embedded within the Al-Ti-Nb-B grain refiner exhibit superior grain refinement efficiency for Al-Si alloys. Their findings suggest that the (0001) crystallographic plane of the (Nb, Ti)B_2_ phase acts as an effective substrate for the epitaxial nucleation of primary α-Al [24]. Furthermore, pioneering research by Li et al. revealed that the addition of titanium (Ti) to Al-Nb-B alloys not only promotes the formation of Al-rich intermetallics but also facilitates the generation of core–shell structured (Nb, Ti)B_2_ particles, with TiB_2_ serving as the nucleus and NbB_2_ forming the outer layer [19]. This study explores the potential of a novel composition in mitigating the adverse effects of Si-rich phases, highlighting a significant interaction between Ti and NbB_2_ during the synthesis of the Al-Ti-Nb-B alloy system.

In this work, a cost-effective Al-Ti-Nb-B composite refining system was developed for HPDC Al-Si alloys by introducing Al-NbB_2_ grain refiners containing micro-nano NbB_2_ particles prepared via a solid–liquid reaction method. Compared with conventional Al-Nb-B or single-element refining approaches, this study systematically investigates the synergistic effects of trace NbB_2_ particles and Ti addition on the microstructure evolution and mechanical performance of HPDC Al-Si alloys. The main contribution of this work lies in clarifying the interaction mechanism between NbB_2_ particles and Ti during grain refinement and demonstrating their effectiveness in improving grain structure and mechanical properties using a relatively low-cost processing route. These findings provide both theoretical insight and practical guidance for the design of high-performance die-cast Al-Si alloys for industrial applications.

## 2. Experimental Procedure

The use of nucleant inoculation (grain refiner) is a crucial technique for achieving grain refinement [25]. In this research, the Al-NbB_2_ master alloy, used as a grain refiner for inoculation, was synthesized via a solid–liquid reaction method. The phase diagram calculations indicated that the reaction within the Al-Nb-B system is thermodynamically favorable for the formation of NbB_2_ particles at a melting temperature range of 800–880 °C, with an optimal temperature of 850 °C being selected for the experiment [26]. A mixture of 20 g of niobium powders and 60 g of KBF_4_ salt was dried and homogenized using a planetary ball mill. This mixture was subsequently encased in aluminum foil and introduced into 400 g of molten aluminum at 850 °C. During a holding period of 2 h prior to casting, manual stirring was conducted for 30 s at 20 min intervals. Post-reaction, the residual by-products were separated from the melt, and the purified melt was cast into preheated molds at 200 °C to produce the Al-NbB_2_ grain refiner. To enable detailed analysis of the particle morphology, the Al-NbB_2_ grain refiner underwent extraction using a 10 vol.% hydrochloric acid solution over a 48 h period [27]. Die-casting experiments were conducted utilizing a 280 ton vertical die-casting machine, where Al-Si series alloys were melted and maintained at 750 °C. Based on previous research, the addition of 0.5 wt.% grain refiner was determined to achieve effective grain refinement. For the present study, we adopted 0.5 wt.% to maintain consistency with previous experiments while ensuring reproducibility of the results under the current processing conditions [28,29]. The Al-NbB_2_ grain refiner was added to the Al-Si alloy melt, and the incorporation of Ti element was facilitated through the addition of an Al-10Ti master alloy. Following the introduction of the grain refiners, the uniformly mixed metal melt was maintained for a duration of 10 min before conducting die casting tests. Three control experiments were performed: one without Al-NbB_2_ and Ti (designated as BC), one with 0.5 wt.% Al-NbB_2_ (designated as NB), and one with both 0.5 wt.% Al-NbB_2_ and 0.1 wt.% Ti (designated as TNB). Although the effect of Ti alone on grain refinement is well-known, our study focuses on the synergistic influence of trace Ti combined with NbB_2_ particles; therefore, a Ti-only control was not included. Table 1 presents the compositions of the Al-Si series alloys for these three experimental conditions. The die-casting process used in this research is shown in Table 2.

To ensure that the microstructural observations of the alloys were representative, samples for microstructural analysis were extracted from the longitudinal center of the cast ingot. After polishing, the samples were etched with Keller’s reagent for 15 to 20 s, and the grain morphology was examined using a Leica DMI5000M optical microscope (Leica Microsystems, Wetzlar, Germany). Quantitative statistical analysis of multiple regions was conducted using ImageJ software (V 6.0) (Bethesda, MD, USA), analyzing a minimum of 100 grains to assess grain size distribution. The microstructural analysis was conducted utilizing an FEI Quanta 650 FEG scanning electron microscope ((FEI Company, Hillsboro, OR, USA) equipped with an electron backscatter diffraction (EBSD) probe and an energy-dispersive X-ray spectroscopy (EDS) system. The EBSD data were subsequently processed using AZtec software (Aztec Crystal 4.0). For a comprehensive examination of the interface morphology, high-resolution transmission electron microscopy (JEOL Ltd., Akishima, Japan) was employed, with TEM samples prepared through focused ion beam (FIB) thinning. The tensile tests were conducted under standard conditions for Al alloys at room temperature, using specimens with geometry consistent with ASTM E8/E8M standards [30]. The tensile properties of the die-cast alloy were evaluated using a WDW-3100 electronic universal testing machine (Jinan Heng Rui Jin Testing Machine Co., Ltd., Jinan, Shandong, China), operating at a loading rate of 1 mm/min. The tensile specimens were characterized by a gauge length of 10 mm, a width and thickness of 3 mm, and an overall length of 28 mm. Each test group comprised a minimum of three specimens, with the average values utilized for analysis.

## 3. Results and Discussion

**Figure 1:**
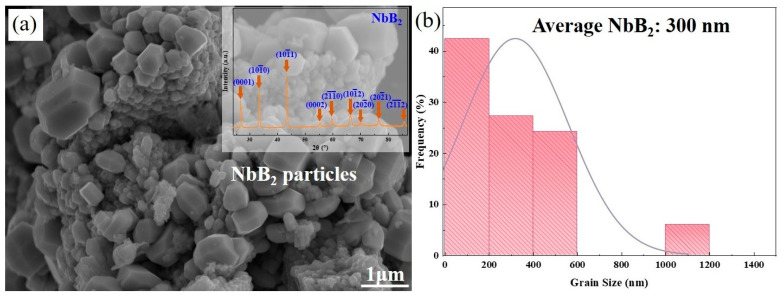
(**a**) Morphology of NbB_2_ particles extracted from the Al-NbB_2_ grain refiner; (**b**) particle size analysis of the NbB_2_ particles.

Figure 1 illustrates the morphology of NbB_2_ particles extracted from the Al-NbB_2_ grain refiner. The X-ray diffraction (XRD) pattern in the upper right corner reveals that the particles exhibit solely NbB_2_ diffraction peaks, indicating that the reaction between Nb powder and KBF_4_ powder in the Al melt was complete. The SEM images reveal that NbB_2_ particles exhibit a typical polyhedral structure, characteristic of the hexagonal crystal structure of NbB_2_. Statistical analysis shows that the particle size of NbB_2_ ranges from 80 nm to 1100 nm, with an average size of 300 nm.

**Figure 2:**
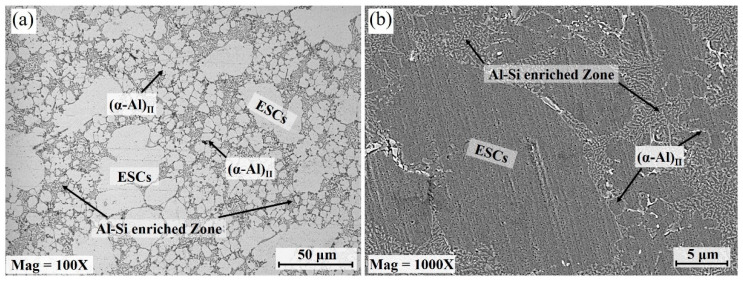
The typical microstructure of die-cast Al-Si series alloy (BC): (**a**) OM image; (**b**) SEM image.

Figure 2 depicts the characteristic microstructure of a die-cast Al-Si series alloy in the absence of grain refinement (BC). The rapid filling and solidification inherent to high-pressure die casting result in the solidified α-Al manifesting as coarse externally solidified crystals (ESCs) and fine secondary α-Al_II_ grains. The ESCs, identified as primary α-Al_I_ grains, exceed 20 μm in size, whereas the α-Al_II_ grains measure less than 5 μm. Importantly, the segregation of Si leads to the presence of Al-Si phases near the Al grain boundaries. This phenomenon occurs because, as the melt enters the die cavity, the lower cavity temperature and rapid cooling rate induce the nucleation of primary α-Al within the die chamber, followed by processes of softening, fragmentation, growth, and migration [11]. Consequently, coarse α-Al grains, known as externally solidified crystals, are formed. Conversely, the strong affinity between Si and Al promotes the formation of Al-Si eutectic phases, which accumulate at the grain boundaries, resulting in Al-Si phase aggregation zones [31]. It is worth noting that the presence of a large number of ESC structures negatively impacts the mechanical properties of the die-cast alloy [32].

**Figure 3:**
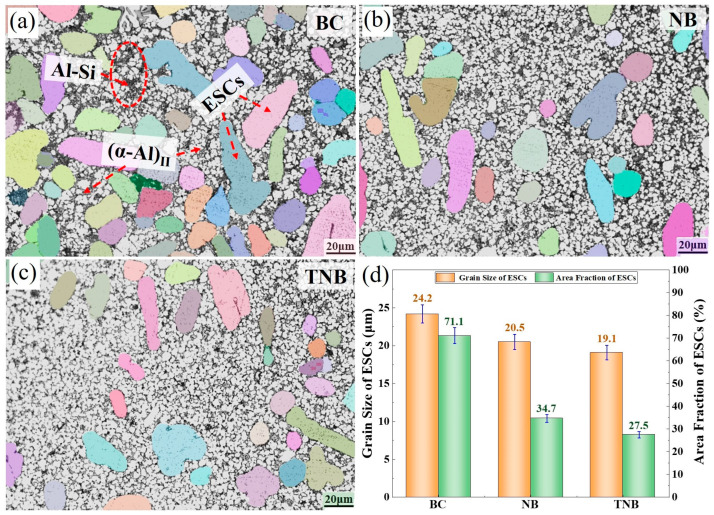
OM images of die-cast Al-Si series alloys with the addition of different grain refiners: (**a**) BC, (**b**) NB, (**c**) TNB, and (**d**) the statistical results of ESC grain size and its area fraction.

Figure 3 presents optical microscopy (OM) images of die-cast Al-Si series alloys both prior to and following the incorporation of various grain refiners. Specifically, Figure 3b and Figure 3c illustrate the Al-Si series alloys with the addition of Al-Nb-B and a composite of Al-Nb-B and Ti, respectively. The findings demonstrate that both Al-Nb-B and Ti are effective in refining the grain structure of the eutectic silicon crystals (ESCs) when compared to the control sample lacking a grain refiner, as evidenced by a marked reduction in grain size and a more regular morphology. Statistical analysis depicted in Figure 3d indicates that the inclusion of NbB_2_ leads to a refinement in ESCs grain size and a decrease in its area fraction. Relative to the benchmark sample, the grain size of ESCs in the NbB_2_-treated sample decreased from 24.2 μm to 20.5 μm, with the area fraction diminishing from 71.1% to 34.7%. Moreover, the combined addition of Al-Nb-B and Ti demonstrated a synergistic effect in grain refinement, surpassing the performance of the singular Al-Nb-B grain refiner. The average grain size of ESCs reached its minimum, decreasing from 24.2 μm to 19.1 μm, and the area fraction was reduced from 71.1% to 27.5%. The changes in grain size and area fraction indicate that with the addition of a grain refiner, the proportion of fine grains in the overall structure significantly increases, with a greater number of grains distributed more uniformly.

**Figure 4:**
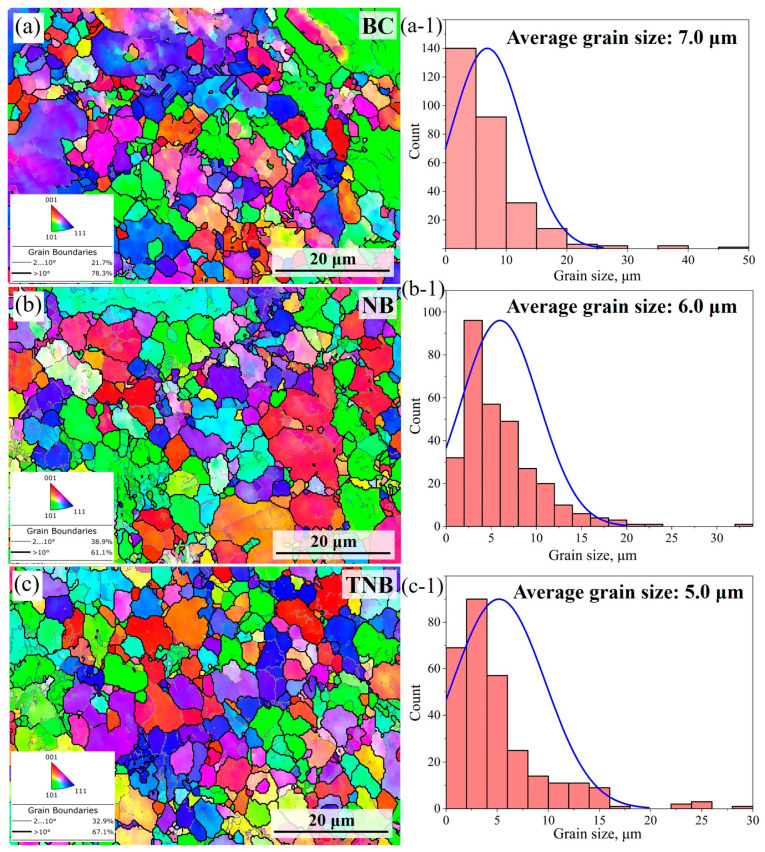
EBSD maps and grain size information of die-cast Al-Si series alloys with the different grain refiners: (**a**) BC, (**a-1**) the grain size information of BC, (**b**) NB, (**b-1**) the grain size information of NB, (**c**) TNB. (**c-1**) the grain size information of TNB.

The quantification of secondary α-Al_II_ grain size in metallographic morphology is challenging due to their smaller dimensions. To further investigate the influence of grain refiners on α-AlII grain size in die-cast Al-Si series alloys, Electron Backscatter Diffraction (EBSD) was utilized to characterize the grain morphology. Figure 4 displays the EBSD images and grain size data for die-cast Al-Si series alloys with various grain refiners. Specifically, Figure 4b and Figure 4c correspond to the Al-Si series alloy with the addition of Al-Nb-B and the combined addition of Al-Nb-B and Ti, respectively. The findings reveal that the addition of Al-Nb-B alone effectively refines the α-Al_II_ grains, decreasing the average grain size from 7 μm to 6 μm. Furthermore, the combined addition of Al-Nb-B and Ti in the die-cast Al-Si series alloy produces a synergistic refinement effect. This composite refiner not only increases the number and uniformity of heterogeneous nucleation sites but also significantly reduces the nucleation energy barrier, resulting in enhanced grain refinement. This microstructure evolution trend suggests that the Al-Nb-B and Ti composite refiner enhances the refinement effect through diversified nucleation mechanisms.

**Figure 5:**
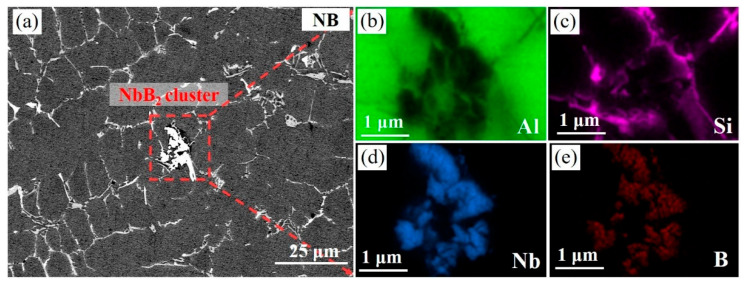
(**a**) Backscattered electron (BSE) images of NB sample; (**b**–**e**) elemental distribution maps of (**a**).

Figure 5 presents backscattered electron (BSE) images of a die-cast Al-Si series alloy containing 0.5 wt.% Al-Nb-B, accompanied by the corresponding elemental distribution maps derived from the BSE images. The images reveal the presence of NbB_2_ particle clusters, approximately 2 to 4 μm in size, within the alloy matrix. Analysis of Figure 5b–e suggests that these clusters consist of NbB_2_ particles. These particles are predominantly located in the central regions of the Al grains, suggesting their potential role in facilitating heterogeneous nucleation of the Al grains. Additionally, there is a notable enrichment of Al-Si phases in proximity to the NbB_2_ particle clusters, indicating that these particles further influence the spatial distribution of Si-rich phases. A comparative analysis of Figure 2 and Figure 5 indicates that the introduction of particles promoting heterogeneous nucleation significantly increases the number of nucleation sites. This results in a higher density of α-Al grain nucleation, which subsequently impedes the growth of large Al-Si eutectic phases, thereby promoting the formation of smaller and more uniformly distributed Al-Si eutectic phases.

**Figure 6:**
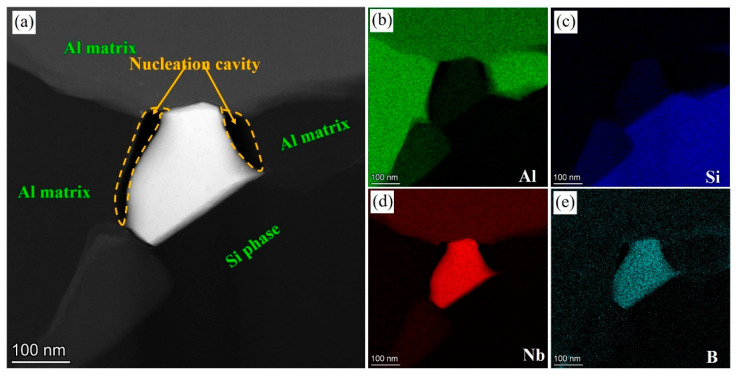
(**a**) TEM images of NbB_2_ particles in NB sample; (**b**–**e**) the elemental distribution maps of Al, Si, Nb, and B in (**a**).

Figure 6 presents transmission electron microscopy (TEM) images of NbB_2_ particles within the NB sample, illustrating particles approximately 200 nm in size embedded in the matrix. The elemental distribution maps of Al, Si, Nb, and B, depicted in Figure 6b–e, confirm the identification of these particles as NbB_2_. Our previous research has demonstrated a favorable lattice matching between Al and NbB_2_, facilitating the preferential nucleation of Al grains on the NbB_2_ substrate [28]. Moreover, according to the E2EM model, there exists a distinct planar mismatch relationship between Al and NbB_2_, specifically (10$\bar{1}$1)/(111), with a spacing mismatch of 3%, which underscores the nucleation potential of NbB_2_ particles [29]. Notably, Figure 6a reveals the presence of nucleation vacancies between the NbB_2_ particle and the Al matrix, which may adversely impact the heterogeneous nucleation performance of NbB_2_ by hindering the direct nucleation of Al grains on NbB_2_. Nevertheless, the incorporation of NbB_2_ particles has resulted in a measurable grain refinement effect.

**Figure 7:**
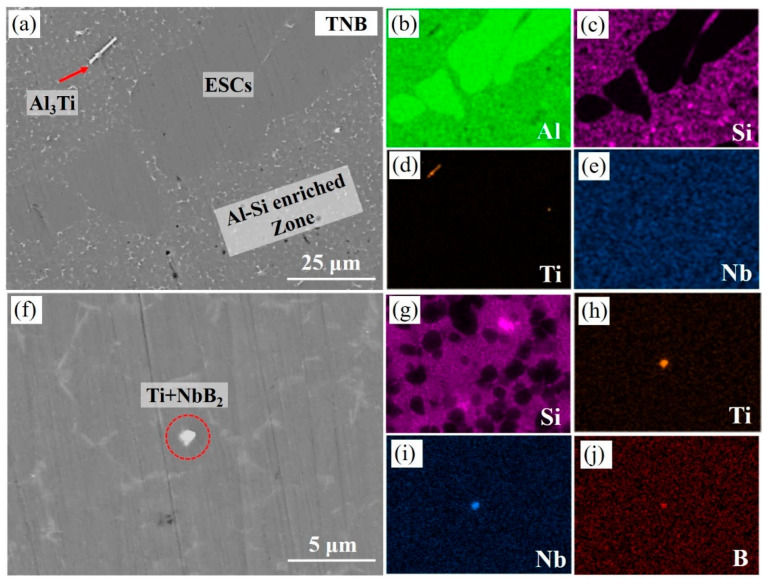
(**a**) SEM images of TNB sample, (**b**–**e**) elemental distribution maps of (**a**), (**f**) particle containing Ti and NbB_2_ particle in TNB sample, and (**g**–**j**) elemental distribution maps of (**f**).

To elucidate the interaction between NbB_2_ particles and the Ti element, a detailed microstructural analysis of the TNB sample was performed. Figure 7 presents the SEM images of the TNB sample, which reveal the presence of irregularly shaped Al_3_Ti phases and particles composed of Ti, Nb, and B elements. Consequently, it can be preliminarily inferred that the addition of Ti not only facilitates the formation of Al_3_Ti with Al but also suggests an interaction between NbB_2_ and Ti, resulting in the formation of Ti-NbB_2_ particles approximately 1 μm in size. The elemental distribution maps in Figure 7g–j indicate that these particles are distributed around the Al-Si phase region. A comparison with Figure 3 reveals that the degree of agglomeration of NbB_2_ particles and the size of the Al-Si phase in the TNB sample are significantly reduced compared to those in the NB sample containing only NbB_2_. This observation suggests that the formation of Ti-NbB_2_ particles, following the interaction between Ti and NbB_2_, effectively suppresses the agglomeration of NbB_2_ and enhances the modification effect on eutectic Si. However, due to the small size of the Ti-NbB_2_ particles, the resolution limitations of SEM preclude a detailed analysis of their crystal structure.

**Figure 8:**
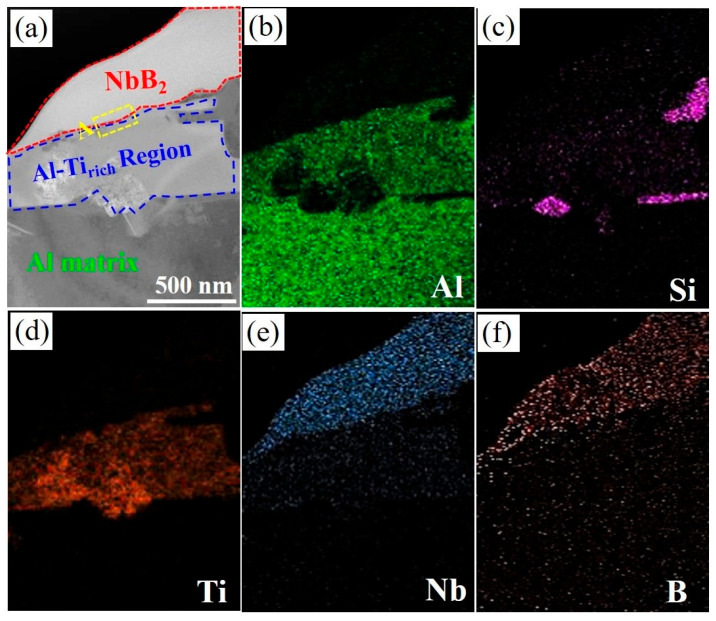
(**a**) TEM morphology of TNB sample containing Ti-NbB_2_ particle picked using FIB techniques; (**b**–**f**) elemental distribution maps of (**a**).

The Ti-NbB_2_ particle depicted in Figure 7f was identified and extracted using focused ion beam (FIB) techniques, followed by the preparation of a TEM sample through ion thinning. Figure 8 illustrates the TEM morphology of the TNB sample containing a Ti-NbB_2_ particle, with energy-dispersive X-ray spectroscopy (EDS) analysis indicating that the structure of the Ti-NbB_2_ particle comprises two components: an NbB_2_ particle and an Al-Ti-rich region. Previous research has identified the presence of a two-dimensional compound (2DC) Al_3_Ti at the TiB_2_/α-Al interface, characterized by the orientation relationship (OR) (0001)_TiB2_//(112)_Al3Ti_-2DC//(111)_Al_. Within this interfacial structure, the formation of Al_3_Ti-2DC occupies the interfacial nucleation vacancies between Al and TiB_2_ particles, thereby reducing lattice mismatch and significantly enhancing the nucleation potential of TiB_2_ for α-Al [17]. Ma et al. further integrated ab initio molecular dynamics (AIMD) and density functional theory (DFT) calculations to investigate the adsorption behavior of Ti at the (0001)_TiB2_/Al interface, revealing a strong adsorption propensity of Ti atoms at the TiB_2_ interface and elucidating its strengthening mechanism [18,33]. The Al-Ti-rich layer at the NbB_2_ interface likely corresponds to an Al_3_Ti-type interfacial compound, similar to the previously reported two-dimensional phase observed at TiB_2_/α-Al interfaces [17]. Literature indicates that Al_3_Ti-2DC formation reduces lattice mismatch and enhances the nucleation potential of TiB_2_ for α-Al. Both NbB_2_ and TiB_2_ exhibit hexagonal crystal structures and possess similar lattice constants, suggesting that Ti may also exhibit strong adsorption on NbB_2_, forming Al_3_Ti layer and ultimately resulting in the formation of Ti-NbB_2_ particles. The TEM results suggest that Ti adsorption at the NbB_2_ interface promotes Al_3_Ti-type layer formation, thereby facilitating the formation and stability of Ti-NbB_2_ particles in the TNB alloy.

**Figure 9:**
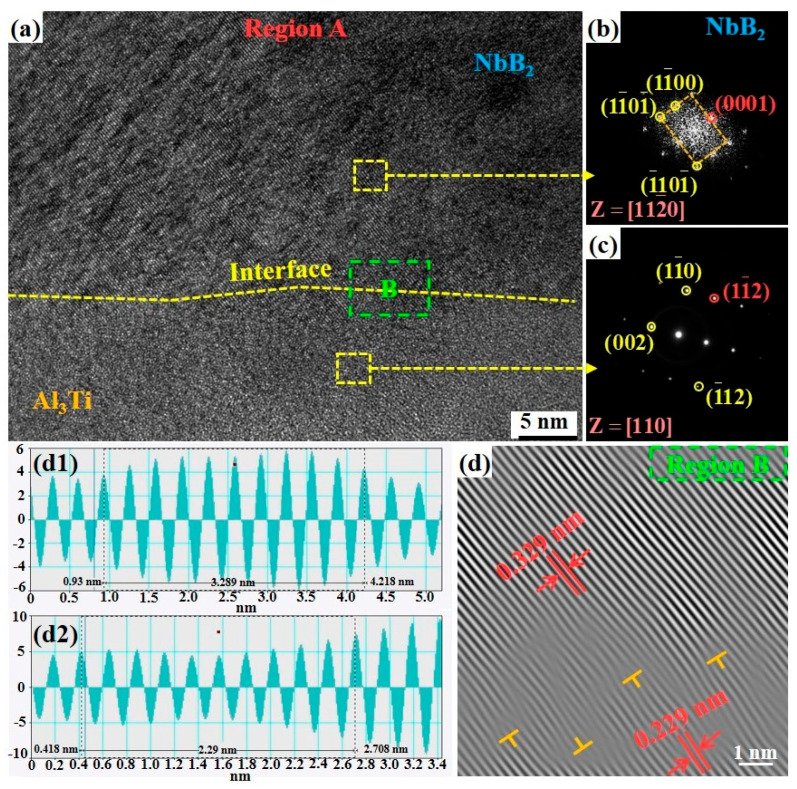
(**a**) HR-TEM morphology of region A in Figure 8a, (**b**,**c**) Fast Fourier transform analysis of the NbB_2_ and Al_3_Ti region, (**d**) Inverse Fast Fourier transform of interface B in (**a**), and (**d1**,**d2**) IFFT statistical analysis of NbB_2_ and Al_3_Ti region.

**Figure 10:**
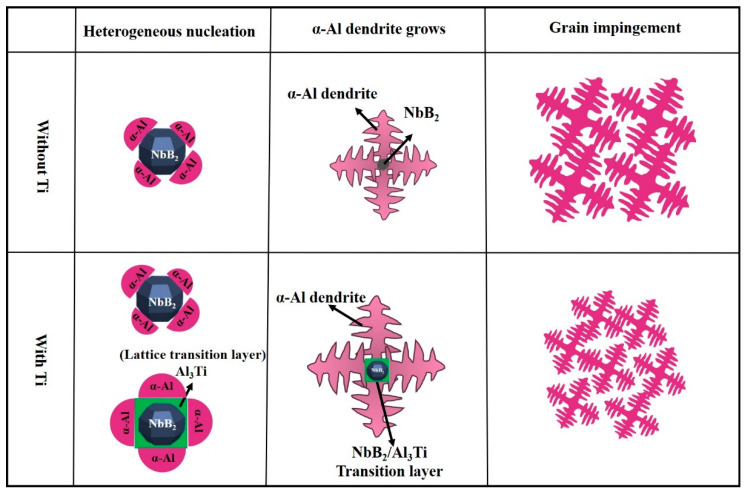
A schematic illustration of the heterogeneous nucleation of NbB_2_ particles relative to Al grains under the influence of Ti element.

To further elucidate the microstructure of Ti-NbB_2_, high-resolution transmission electron microscopy (HR-TEM) analysis was performed on region A, as delineated by the red box in Figure 8a. Figure 9a illustrates the HR-TEM morphology of region A, highlighting the NbB_2_ particle situated above and the Al-Ti-rich region below. Fast Fourier Transform (FFT) analysis of the NbB_2_ region is presented in Figure 9b, which reveals its crystal zone axis and confirms its hexagonal close-packed (HCP) structure, corresponding to the P6/mmm space group, thereby verifying the identification of this region as NbB_2_. Similarly, the Al-Ti-rich region was subjected to selected area electron diffraction (SAED), depicted in Figure 9c, with the diffraction spots corroborating its tetragonal structure, associated with the I4/mmm space group, thus identifying this region as Al_3_Ti. Further examination of the interface matching conditions was conducted using Inverse Fast Fourier Transform (IFFT), as illustrated in Figure 9d. A considerable number of lattice distortions and edge dislocations are evident at the interface. Statistical analysis indicates that the interplanar spacing of (0001)_NbB2_ is 0.329 nm, while that of (1$\bar{1}$2)_Al3Ti_ is 0.229 nm, further substantiating that these two regions correspond to a matching transition layer between NbB_2_ and Al_3_Ti. Figure 10 shows a schematic illustration of the heterogeneous nucleation of NbB_2_ particles relative to Al grains under the influence of Ti element. The introduction of Ti can form a transition layer between Al and NbB_2_. The presence of the transition layer can be inferred to not only occupy the interface nucleation vacancies between NbB_2_ and Al, thereby facilitating the nucleation of Al atoms at their interface, but also to establish a precise matching relationship between the interface layers. This enhances their heterogeneous nucleation effect on the Al matrix and further improves the refinement performance of the Al-Nb-B grain refiner.

**Figure 11:**
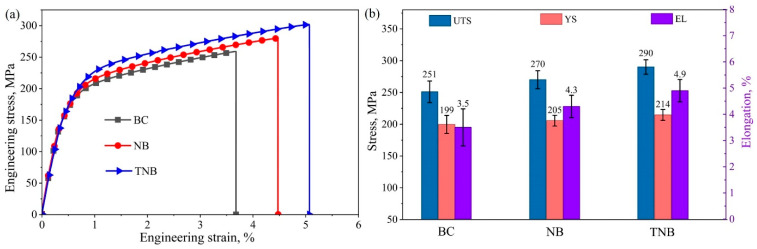
(**a**) Engineering stress–strain curves of the die-cast Al-Si series alloys; (**b**) a summary of mechanical performance data.

**Figure 12:**
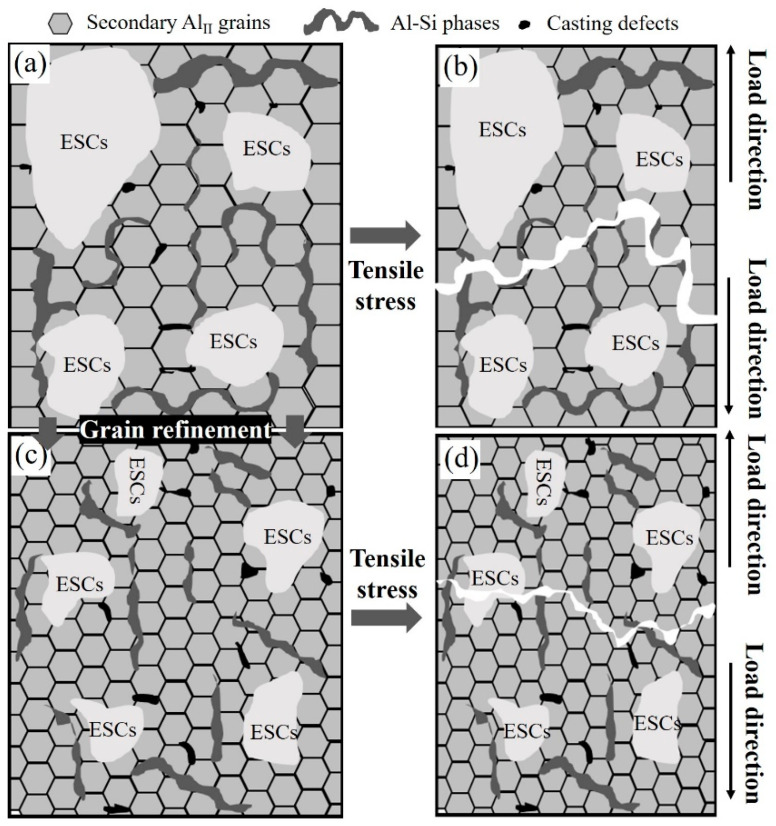
The evolution of fracture patterns in Al-Si series alloy during tensile testing: (**a**,**b**) original state Al-Si series alloy; (**c**,**d**) Al-Si series alloy with grain refinement.

Figure 11 presents the engineering stress–strain curves and summarizes the mechanical performance data for die-cast Al-Si series alloys. In the BC sample, the presence of coarse eutectic silicon crystals (ESCs) and large α-Al_II_ grains results in a yield strength of 199 MPa, a tensile strength of 251 MPa, and limited plasticity of approximately 3.5%. Following the inoculation with Al-Nb-B, the ESCs and α-Al_II_ grains are refined, leading to an increase in the yield strength of the Al-Si series alloy to 205 MPa, a tensile strength of 270 MPa, and an elongation of 4.3%. When Al-Nb-B is combined with the Ti element, the sizes of the ESCs and α-Al_II_ grains are further reduced, resulting in more uniform morphologies and distribution of the Al-Si phase. The composite refining effect significantly enhances the mechanical properties of the alloy, achieving a yield strength of 214 MPa, a tensile strength of 290 MPa, and an elongation of 4.9%. Figure 12 illustrates the evolution of fracture patterns in Al-Si series alloys during tensile testing, both with and without grain refinement. In the absence of a grain refiner, cracks preferentially propagate along coarse ESCs, large α-Al grains, and regions with casting defects such as porosity and inclusions, which limits both strength and ductility. With grain refinement, the reduction in grain size and ESC dimensions decreases defect-related stress concentration, shifting crack paths to traverse finer α-Al regions and ESCs. This change in fracture morphology, together with the more homogeneous phase distribution, explains the simultaneous improvement in strength and elongation [34,35]. The fraction of cracks propagating through ESCs versus α-Al regions decreases with refinement, while porosity-related crack initiation is significantly reduced. Overall, these results demonstrate a clear microstructure–mechanical property correlation, highlighting that NbB_2_ and Ti-induced refinement strengthens the alloy through a combination of Hall–Petch strengthening and suppression of defect-driven fracture, as depicted in Figure 12.

## 4. Conclusions

This study elucidates the synergistic refinement and strengthening mechanisms induced by trace additions of NbB_2_ and Ti in high-pressure die-cast (HPDC) Al-Si alloys. The mechanisms underlying the evolution of microstructure and mechanical performance are systematically examined. The principal conclusions are as follows:

(1) The incorporation of NbB_2_ results in significant reductions in both the size and area fraction of eutectic silicon crystals (ESCs) and secondary α-Al grains, while also enhancing the microstructural uniformity and mechanical performance of the Al-Si alloy series. Furthermore, the synergistic interaction with titanium (Ti) further amplifies the refining efficacy of NbB_2_ particles. The alloy exhibiting the combined addition of Ti and NbB_2_ achieves optimal refinement outcomes, characterized by an ESC grain size of 19.1 μm, an area fraction of 27.5%, and an α-Al grain size of 6 μm.
(2) The synergistic addition of Ti and NbB_2_ facilitates the formation of Ti-NbB_2_ hybrid particles, accompanied by the development of a Ti-rich transition layer at the interface between NbB_2_ and aluminum. This transition layer not only mitigates interface nucleation vacancies, thereby promoting the nucleation of aluminum atoms at the interface, but also establishes a compatible relationship between the two phases, further enhancing the refinement performance.
(3) The mechanical properties of the modified alloy are substantially improved, demonstrating notable enhancements in yield strength, tensile strength, and elongation. The incorporation of the Ti element with Al-Nb-B further reduces the sizes of the eutectic silicon crystals (ESCs) and α-Al_II_ grains, while also promoting a more uniform morphology and distribution of the Al-Si phase. Consequently, the Al-Si series alloy, with the synergistic addition of Al-Nb-B and Ti, achieves optimal mechanical properties, characterized by a yield strength of 214 MPa, a tensile strength of 290 MPa, and an elongation of 4.9%. These improvements are attributed to the refined grain structure and the minimization of casting defects.

## Figures and Tables

**Table 1:** The composition of the alloy under the three sets of experiments (wt.%).

Alloy	Si	Mg	Cu	Mn	Fe	Zn	Ti	Al-NbB_2_	Al
BC	7.15	0.43	0.32	0.01	0.13	0.01	0	0	Bal.
NB	6.82	0.47	0.25	0.01	0.14	0.01	0	0.5	Bal.
TNB	6.95	0.44	0.28	0.01	0.12	0.01	0.11	0.5	Bal.

**Table 2:** The Al-Si alloy die-casting process parameters used in this research.

Parameters	Injection Speed	Specific Pressure of Injection	Casting Temperature	Mould Temperature
Value	300 mm/s	170 MPa	690 °C	220 °C

## Data Availability

The original contributions presented in the study are included in the article, further inquiries can be directed to the corresponding author.
